# A Huge Intra‐Abdominal Mesenteric Cyst in a Child Mimicking Ascites: A Case Report

**DOI:** 10.1002/ccr3.73133

**Published:** 2026-07-10

**Authors:** Samina Chaki, Abizer Sarkar, Ronald Mclarty, Rawya Baabde, Hilary Chipongo, Evelyne Assenga, Karim Manji

**Affiliations:** ^1^ Department of Pediatrics and Child Health Muhimbili University of Health and Allied Sciences Dar es Salaam Tanzania; ^2^ Department of Radiology Shree Hindu Mandal Hospital Dar es Salaam Tanzania; ^3^ Department of Pediatrics and Child Health Shree Hindu Mandal Hospital Dar es Salaam Tanzania; ^4^ Department of Anatomical Pathology Muhimbili University of Health and Allied Sciences Dar es Salaam Tanzania; ^5^ Department of Pediatrics and Child Health Muhimbili National Hospital Dar es Salaam Tanzania

**Keywords:** ascites, mesenteric cyst, pediatrics, peritoneum

## Abstract

This is a unique case of a suspected huge mesenteric cyst in a pediatric patient presenting as an ascitic process. The patient had an insidious clinical presentation and had no predisposing factors. Mesenteric cysts are extremely rare in pediatrics.

## Key Clinical Message

Mesenteric cysts, although rare in pediatric patients, can present with nonspecific symptoms and mimic ascites, making diagnosis challenging. Awareness of this possibility is important to avoid misdiagnosis and ensure timely surgical management, which typically results in excellent outcomes.

**Abbreviations**
CT‐SCAN: computed tomography
MC: mesenteric cyst
RR: respiratory rate

## 1 Introduction

Mesenteric cysts are rare intra‐abdominal lesions that often present with few or nonspecific symptoms, making preoperative diagnosis challenging [1]. They are typically identified using abdominal ultrasound or computed tomography. The differential diagnosis includes a variety of abdominal and retroperitoneal masses. Definitive management is surgical, and complete excision of the cyst can be performed either via laparotomy or laparoscopic approaches [1]. Although the origin of mesenteric cysts is uncertain, several developmental theories have been proposed [2]. Complete surgical removal remains the preferred treatment, and since these lesions are rare and often lack specific clinical features, establishing an accurate preoperative diagnosis is challenging [3]. Awareness of mesenteric cysts is essential, as inadequate management can lead to complications [1]. Imaging studies such as ultrasound and computed tomography are helpful but may still be misleading, especially when the cyst resembles free intraperitoneal fluid [4]. We report a case of a large mesenteric cyst with atypical presentation that mimicked ascites.

## 2 Case History and Examination

This is a 6‐year‐old male patient who presented to the clinic with a complaint of abdominal swelling and early satiety for more than 3 months. The condition was associated with episodes of vomiting, which were non‐projectile, and occasional episodes of on‐and‐off difficulty in breathing. No history of trauma or any other predisposing factor was reported. On the past medical and social history, there were no significant findings. On examination, the abdomen was distended with a positive fluid thrill test and shifting dullness. He was slightly tachypneic with RR 24 cycles per minute; other vitals were within normal range for his age.

## 3 Investigations and Management

**FIGURE 1:**
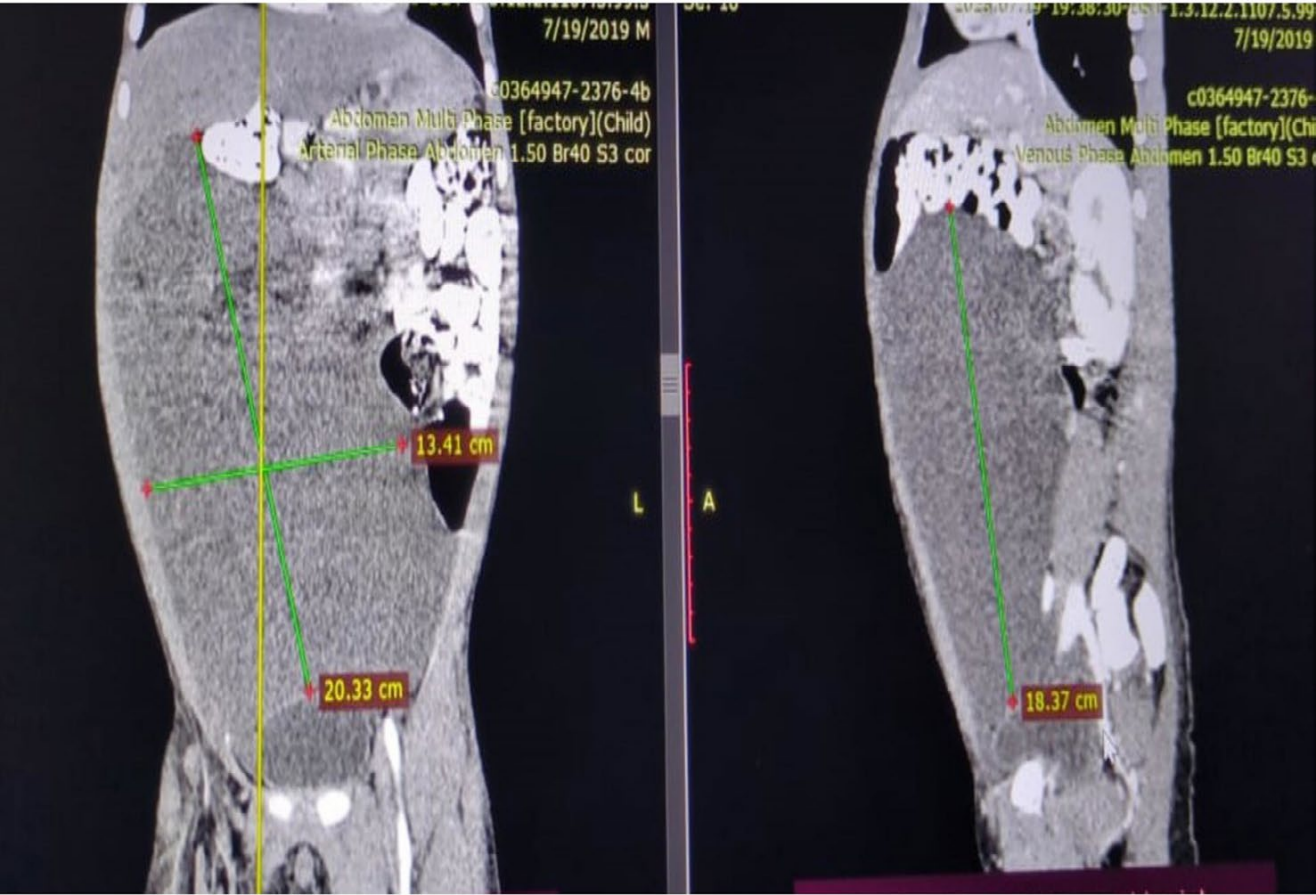
CT‐Scan revealed a huge mesenteric cyst measuring approximately 20 × 18 × 13 cm in size with fluid contents.

**FIGURE 2:**
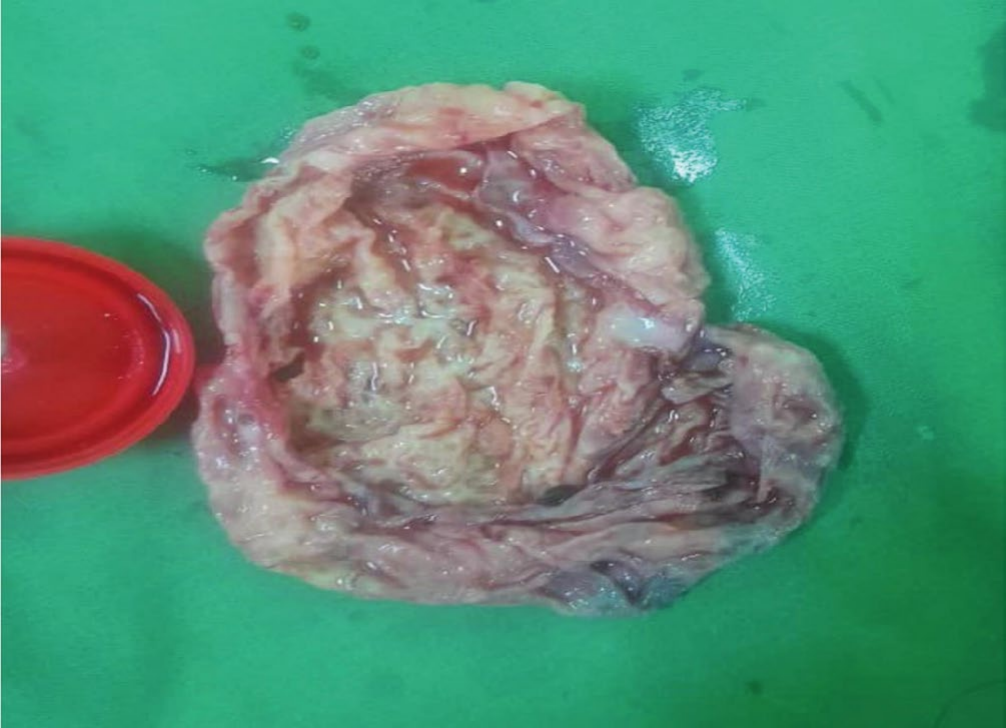
Gross appearance of the cystic lesion.

**FIGURE 3:**
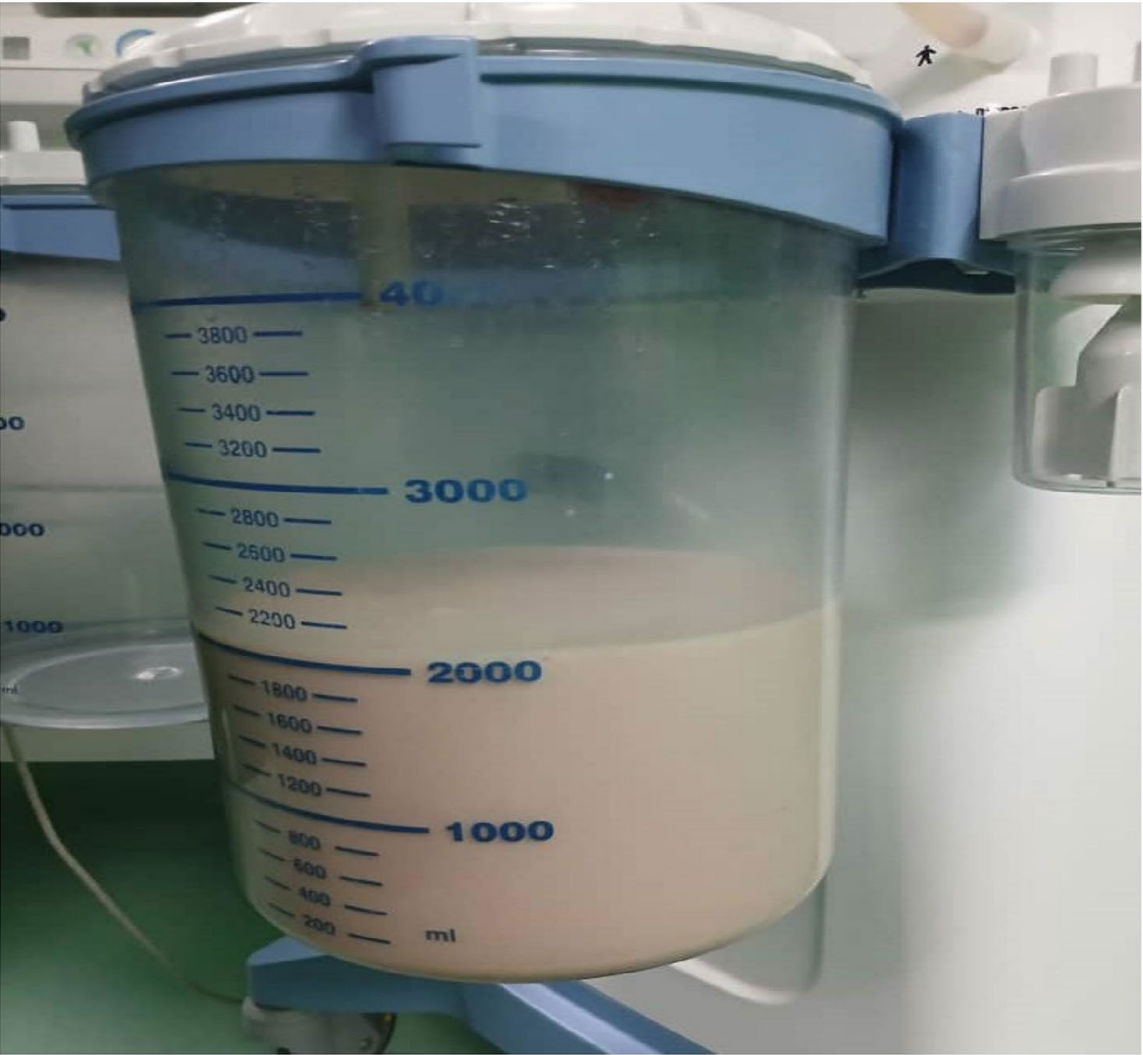
Milky‐white contents suctioned from the lesion.

Laboratory investigations done were unremarkable. Cross‐sectional imaging done (CT‐Scan) revealed a huge mesenteric cyst measuring approximately 20 × 18 × 13 cm in size with fluid contents (see Figure 1). The patient was scheduled for cystectomy, which was performed, confirming the cystic lesion, which is shown in Figure 2. During the procedure, the lesion contained approximately 2 L of milky white fluid resembling chylous ascites (see Figure 3).

## 4 Results

Following surgery, the patient experienced an uneventful 4‐week postoperative period.

## 5 Discussion

Mesenteric cysts represent an uncommon group of benign intra‐abdominal lesions that often remain diagnostically elusive due to their rarity and lack of characteristic clinical features [1]. Their incidence is low, and as a result, they are not frequently considered early in the differential diagnosis of patients presenting with abdominal distension or pain [5]. This case underscores the diagnostic ambiguity associated with mesenteric cysts, particularly when they present with features closely resembling ascites, as occurred in our patient.

Clinical manifestations of mesenteric cysts are highly variable and are largely influenced by cyst size, anatomical location, and the presence of secondary complications [6]. While smaller cysts may remain clinically silent, larger lesions often produce symptoms through mass effect, including abdominal discomfort, progressive distension, early satiety, or bowel‐related symptoms [5, 6]. Limited cases of pediatric patients with mesenteric cysts have been documented in the literature. This is a unique case of a child who presented with abdominal swelling and early satiety, without systemic features or signs of bowel obstruction. This nonspecific presentation contributed to the initial diagnostic uncertainty and highlights why mesenteric cysts are frequently mistaken for more common conditions such as ascites.

Radiological evaluation is central to the diagnostic work‐up of suspected mesenteric cysts. Ultrasonography is widely used as an initial imaging modality due to its accessibility and non‐invasive nature; however, large cystic lesions may appear indistinguishable from free intraperitoneal fluid [1, 7]. Cross‐sectional imaging with computed tomography provides superior anatomical detail and may assist in identifying a well‐circumscribed cystic structure arising from the mesentery [7]. Nevertheless, even advanced imaging techniques may fail to establish a definitive diagnosis preoperatively, particularly in giant cysts that mimic ascites or multiloculated fluid collections. Similar diagnostic challenges have been documented in previously reported cases of giant mesenteric cysts [8].

The exact etiology of mesenteric cysts remains a subject of debate. Proposed mechanisms include congenital maldevelopment of lymphatic channels, failure of lymphatic drainage into the venous system, trauma, infection, or degenerative changes within mesenteric lymph nodes [5]. Most lesions are believed to be lymphatic in origin and are histologically classified as lymphangiomas. Regardless of the underlying pathogenesis, definitive diagnosis is usually achieved only after surgical excision and histopathological examination [9]. In our case, histology was not performed due to financial factors; the diagnosis was entirely radiologically, grossly, and clinically based. CT‐Scan features, such as thin‐walled, water‐attenuated lesions, which are frequently located in the small bowel with the asymptomatic presentation of abdominal pain, distension, a palpable mass, or vomiting such as in our patient, aided the provisional diagnosis.

Surgical intervention remains the cornerstone of management for mesenteric cysts. Complete excision is recommended whenever feasible, as it minimizes the risk of recurrence and allows for definitive histological confirmation [9, 10]. The choice of surgical approach depends on cyst size, location, and involvement of adjacent structures [11]. Although minimally invasive techniques have gained popularity, open surgery may still be required in cases of large cysts or when there is concern for bowel involvement. In our patient, complete excision resulted in full resolution of symptoms, reinforcing the effectiveness of surgical management.

Failure to recognize and treat mesenteric cysts promptly may lead to complications such as infection, hemorrhage, rupture, torsion, or intestinal obstruction. Rare cases of malignant transformation have also been described, further supporting the need for definitive surgical treatment [5]. The favorable postoperative course observed in this case is consistent with existing literature, which reports excellent outcomes following complete excision and low rates of recurrence [9, 10, 11].

This case emphasizes the importance of maintaining a broad differential diagnosis when evaluating patients with unexplained abdominal distension and pain. In particular, clinicians should consider mesenteric cysts when imaging findings are atypical for ascites or when the clinical picture does not fully align with more common etiologies. Heightened awareness of this rare entity may facilitate earlier diagnosis, prevent unnecessary delays in management, and reduce the risk of complications.

In summary, mesenteric cysts are rare lesions that may present with nonspecific symptoms and closely mimic ascites, posing a significant diagnostic challenge. Imaging studies are invaluable but not always conclusive, and surgical exploration often remains necessary for definitive diagnosis and treatment. Complete excision offers excellent prognosis and should be pursued whenever possible. In resource‐limited settings where histological examination is limited, a high suspicion index should always be considered in patients presenting with ascites, especially in pediatric populations.

## Author Contributions

**Samina Chaki:** conceptualization, writing – original draft. **Ronald Mclarty:** data curation, methodology. **Rawya Baabde:** data curation. **Evelyne Assenga:** validation. **Abizer Sarkar:** methodology, writing – review and editing. **Hilary Chipongo:** supervision. **Karim Manji:** supervision.

## Funding

The authors have nothing to report.

## Disclosure

Guarantor: I, Dr. Hilary Chipongo.

## Ethics Statement

This study is exempt from ethical approval as per our institution's guidelines, as it involves a single patient case that is anonymized and does not include any identifiable personal information.

## Consent

Written informed consent was obtained from the patient's guardian for publication and any accompanying images. A copy of the written consent is available for review by the Editor‐in‐Chief of this journal on request.

## Conflicts of Interest

The authors declare no conflicts of interest.

## Data Availability

The data used to support the findings of this study are available from the corresponding author upon reasonable request.

## References

[1] L. A. Barbu , N. D. Mărgăritescu , L. Cercelaru , et al., “Mesenteric Cysts as Rare Causes of Acute Abdominal Masses: Diagnostic Challenges and Surgical Insights From a Literature Review,” Journal of Clinical Medicine 14, no. 14 (2025): 4888. 40725581 10.3390/jcm14144888 PMC12295725

[2] S. Y. Guraya , S. Salman , and H. H. Almaramhy , “Giant Mesenteric Cyst,” Clinical Practice 1, no. 4 (2011): e108, 10.4081/cp.2011.e108. PMC3981406 24765349

[3] A. B. Shewaye , K. A. Berhane , A. G. Gebresilassie , et al., “Giant Mesenteric Cyst in a Young Adult Mimicking Refractory Ascites: A Diagnostic and Surgical Challenge‐A Case Report,” Case Reports in Gastrointestinal Medicine 2025 (2025): 7405161. 40313350 10.1155/crgm/7405161 PMC12043438

[4] J. H. Yacoub , J. A. Clark , E. E. Paal , and M. A. Manning , “Approach to Cystic Lesions in the Abdomen and Pelvis, With Radiologic‐Pathologic Correlation,” Radiographics 41, no. 5 (2021): 1368–1386. 34469214 10.1148/rg.2021200207 PMC8415047

[5] A. K. Pithawa , A. S. Bansal , and S. P. Kochar , “Mesenteric Cyst: A Rare Intra‐Abdominal Tumour,” Medical Journal, Armed Forces India 70, no. 1 (2014): 79–82. 24936122 10.1016/j.mjafi.2012.06.010 PMC4054796

[6] K. Kaushik , A. Pratap , B. Naik , A. Datta Sai Subramanyam , and M. A. Ansari , “Intact Excision of a Mesenteric Pseudocyst,” Cureus 15 (2023): e40615. 37476128 10.7759/cureus.40615 PMC10354564

[7] S. F. Crinò , L. Bernardoni , E. Manfrin , A. Parisi , and A. Gabbrielli , “Endoscopic Ultrasound Features of Pancreatic Schwannoma,” Endoscopic Ultrasound 5 (2016): 396–398, 10.4103/2303-9027.195873. 28000633 PMC5206830

[8] M. Vallejo‐Soto and S. Orozco‐Simental , “A Giant Mesenteric Cyst Mimicking Untreatable Ascites,” Revista de Gastroenterología de México 82, no. 4 (2017): 348–351. 28215472 10.1016/j.rgmx.2016.04.006

[9] M. Alqreea , M. Sleiay , H. Haydar , M. O. Khedr , J. Z. Damlakhi , and A. M. Kanaan , “Diagnosis and Surgical Management of Mesenteric Cysts in a 10‐Year‐Old Female Patient: A Case Report,” International Journal of Surgery Case Reports 129 (2025): 111108. 40056810 10.1016/j.ijscr.2025.111108 PMC11930728

[10] B. T A, Kuladeep , A. Sujay Mendon , and K. PH , “Cyst in the Mist: A Surgical Perspective on Mesenteric Cysts,” Cureus 17, no. 7 (2025): e88074. 40821161 10.7759/cureus.88074 PMC12356163

[11] N. A. Alenazi , K. S. Ahmed , M. S. Essa , W. I. Abusiam , and A. M. Al‐Shoaibi , “Laparoscopic Excision of Large Mesenteric Cyst From the Small Bowel Mesentery in Adult Male Patient,” International Journal of Case Reports and Images 10 (2019): 1–5.

